# The Pharmacopœia of the United States of America

**Published:** 1851-07

**Authors:** 


*The Pharmacopœia of the United States of America.* By Authority of the National Medical Convention, held at Washington, A. D., 1850. Philadelphia: Lippincott, Grambo & Co.

A new edition of the United States Pharmacopœia, as our readers doubtless are aware, is issued at the expiration of every ten years, after a revision by a convention of delegates from the State Medical Societies and Medical Colleges of the country. The decennial Convention assembled at Washington, in May, 1850, and, as the result of their proceedings, the fourth revised edition of the Pharmacopœia is now published.

With respect to the alterations and improvements of the present edition, we will take the liberty of borrowing the remarks of a reviewer in our esteemed contemporary the New Jersey Medical Reporter:

"An object which seems to be kept constantly in view, is to embrace in the Pharmacopœia, only really valuable and well established medicines. Among the multitude of new remedies, whether drugs or preparations, which are constantly rising to the surface, as it were, of that vast chaotic mass of materials called the materia medica, not a few attract a temporary notice, and, in a very few years, sink again into oblivion. If every article of this kind were incorporated into the Pharmacopœia as soon as introduced, it is obvious that we should cumber each new edition of the work with a variety of articles which would have to be stricken out in the next. Partly with a view to obviate this difficulty, and also to confine the officinal list within narrower bounds than would otherwise be possible, the Pharmacopœia of the United States contains a secondary list which embraces a variety of drugs, not very often prescribed, yet having been at one time in vogue, or being in use perhaps in a limited section of country, or being indigenous to our own country, and little known to practitioners, require some notice. In the new edition, we find four substances transferred from the secondary to the primary list, as having attained a higher reputation during the past ten years; these are Bromine, Irish Moss (Chondrus,) Marsh Rosemary (Statice,) and Queen's Root (Stillingia.) The drugs made officinal for the first time are sixteen in number; among them the most important are Aconite Root, Cod Liver Oil, (Oleum Morrhuæ,) Oil of Bitter Almonds, Brandy, (Spiritus Vini Gallici,) and Port Wine, (Vinum Rubrum.) In the above remark reference has been had to the materia medica list, as distinguished from the preparations. This consists merely of a list of recognized drugs found in commerce, with their proper names, sources, and tests of purity.

The preparations occupy by far the larger part of the work, comprising formulæ for all the recognized medicinal compounds prepared by the apothecary, with such descriptions of them as will direct the physician or pharmaceutist in judging of their quality and strength when prepared. It was in revising this portion of the work that by far the greatest amount of labor and practical skill was requisite, and the greatest room allowed for addition and improvement. Yet here, as in the list, great care seems to have been taken not to add anything which is not absolutely called for by the existing state of pharmacy. While it is desirable to have authoritative formulæ in all instances in which variations in strength would be followed by serious inconvenience, great caution must be used in giving the sanction of the law to any preparation having a mere local or circumscribed reputation, or which is not likely to become a general remedy. The multiplication of officinal formulæ for compounds has direct tendency to circumscribe, rather than to enlarge, the number and variety of medicinal preparations; by saving the practitioner the trouble of contriving an extemporaneous compound, exactly adapted to the case, it presents him a temptation to use one which, though perfect in itself, cannot be considered perfectly adapted to all the variations in disease. Hence the U. S. Pharmacopœia contains few mixtures, pills, or compound powders, these being left mainly to extemporaneous prescription.

There are, however, fifty-three new preparations introduced into the present edition: of these some are chemical, and some tinctures, syrups and extracts. We also have a new class of preparations, for the first time recognized as officinal—the fluid extracts. This is one of the most desirable improvements that has been made; though we cannot but regret that preparations so very distinct in their characters, as fluid extracts of senna (a concentrated syrup,) and fluid extract (oleo resin) of cubebs, should be classed under the same general head. Donovan's solution, solution of nitrate of iron, and solution of citrate of magnesia, will be welcomed, especially as the formulæ for preparing them are distinguished by great simplicity and neatness. A further notice of the new preparations would perhaps hardly be appropriate here, as it would extend the limits of this notice beyond the allotted space.

Before concluding, however, it is proper to remark upon the general execution of the book:—The printing is done in the very best manner, upon remarkably fine paper, and the whole is furnished in a neat and substantial binding, as it should be. To this, however, we feel constrained to add, that the publishers have issued but a small edition, at what we consider an exorbitant price. That this is just cause of complaint, will appear when we state that the whole number of pages is 317, including preliminary notices and all; it contains no illustrations, the margins are wide, the type large, and yet it is sold at $2 50 per copy. It ought to be a cheap book, and widely diffused. We also desire to call attention to two or three slight errors which have escaped the proof-readers. In the formula for tincture of ginger by the displacement process, the term diluted alcohol is used instead of alcohol. In the formula for compound syrup of sarsaparilla by displacement, page 228, the term *two* pints is used instead of *ten* pints. This is corrected in part of the edition. In the preface, page xvii, Quercus Tinctoria is spelled Tinctorea, and on the next page Fœniculum is spelled Fœniculun. Considering the great care taken in the revision of this work, and the number and character of the proof-readers engaged in it, these mistakes may be considered as indicating the impossibility of reaching perfection in any human undertaking." E. P.

The advantages of a standard national Pharmacopœia are too obvious to require remark, and it is quite unnecesary to commend the work to the patronage of the medical profession of the United States.

